# No Effect of *Bt-*transgenic Rice on the Tritrophic Interaction of the Stored Rice, the Maize Weevil *Sitophilus Zeamais* and the Parasitoid Wasp *Theocolax elegans*

**DOI:** 10.1038/s41598-019-40836-8

**Published:** 2019-03-13

**Authors:** Qingfeng Tang, Zhipeng Yang, Rongrong Han, Ying Zhang, Chen Shen, Jian Wang

**Affiliations:** 1 Department of Entomology, College of Plant Protection, Anhui Agricultural University, Hefei, Anhui 230036 China; 2 Department of Entomology, University of Maryland, College Park, MD 20742 USA

## Abstract

During *Bt* transgenic rice storage, *Bt* Cry1Ab/Cry1Ac fused protein is exposed to the maize weevil *Sitophilus zeamais* and the parasitoid wasp *Theocolax elegans*. We have carried out a long-term risk assessment for *Bt* rice to these non-target organisms in the storehouse. Effects of *Bt* rice on *S*. *zeamais* and *T*. *elegans* have been carefully detected in a laboratory experiment of over 5 years. The survival, development, fecundity, and longevity of the maize weevil were compared between *Bt* rice and non-*Bt* rice treatments for every 5 generations from generation 1 to 25. Moreover, the development, adult body size and sex ratio of *T*. *elegans* were compared between them parasitizing *S*. *zeamais* feeding on *Bt* rice or non-*Bt* rice. We found that although *Bt* Cry1Ab/Cry1Ac fused protein exists in the *Bt* rice grains and *S*. *zeamais* digestive tracts, *Bt* rice is not harmful to the maize weevil *S*. *zeamais* and its parasitoid *T*. *elegans*.

## Introduction

Rice (*Oryza sativa* L) is one of the most important grain crops. It serves as a staple diet for more than half of the world’s population^1^. The major insect pests of rice production are hemipteran sap-sucking planthoppers and lepidopteran stem borers and leaf folders. The latter includes yellow stem borer (*Scirphophaga incertulus* Walker), striped stem borer (*Chilo supressalis* Walker), pink stem borer (*Sesamia inferens* Walker), and leaf folder (*Cnaphalocrosis medinalis* Guenee), which cause severe yield losses annually^2^. Every year, insecticides have been extensively applied to control these lepidopteran pests during the commercial rice production, which is expensive and environmentally unfriendly.

With wide use of modern biotechnology in rice breeding programs, many foreign genes have been introduced into rice to generate genetically modified (GM) rice varieties that resist these lepidopteran pests^3,4^. Among them, the *Bacillus thuringiensis* crystal (*Bt* Cry) insecticidal protein (δ-endotoxin) genes have shown good performance in public and private sector rice breeding programs^5^. These *Bt*-crystal genes are highly selective and represent a class of numerous proteins with insecticidal action on larvae from various orders. For example, Cry1 and Cry2 are toxic to lepidopteran pests, specially^6^. So far, rice has been genetically transformed with genes of *Bt cry1Ab*, *cry1Ac*, *cry1Ca*, *cry2A* and *cry1Ab*/*cry1Ac* fusion to control these stem borers and leaf folders^7^. Field study with a *Bt* rice line in China revealed an increase in yield by 6–9% and a reduction in pesticides usage by 80%^8^.

However, before a *Bt* rice line can be cultivated, the risks to the environment and human health must be assessed. This includes the evaluation of potential adverse effects on non-target invertebrate and vertebrate animals, as well as the ecosystem services they provide^9–12^. For this purpose, extensive investigations have been conducted in the rice field. First, several studies focused on the effects of *Bt*-transgenic rice lines on the non-target arthropods in the rice field were performed. These studies revealed that *Bt*-transgenic rice does not directly affect the growth, development, abundance, and diversity of brown planthopper^13–15^, leafhopper^13^, ground-dwelling collembolans^16^, pond wolf spider and other rice field predator natural enemies^13,17–20^. Second, biosafety of *Bt*-transgenic rice to agroecosystems, including soil and aquatic ecosystem, was assessed. It was reported that Cry1Ab and Cry1Ac toxins from *Bt* rice plant tissues could persist in paddy fields for a long period of time^21^. These Cry proteins could accumulate further in the soil by binding to soil particles and remain biologically active^22–24^. However, transgenic *Bt* rice is safer to aquatic ecosystems than its non-transgenic counterpart. The abundance and diversity of zooplankton were significantly higher in *Bt* rice fields than non-*Bt* rice fields, because *Bt* rice required reduced pesticide application therefore produced lower pesticide residues in the field water body^25,26^. Finally, safety of *Bt*-transgenic rice to the vertebrates were investigated. Impact of *Bt* transgenic rice on frogs^27,28^, rats^29–32^, pigs^26^, and macaques^33^ was intensively studied. Through a 90-days feeding assay, no differences were observed between the rats feeding on genetically modified rice and non-genetically modified rice in term of a range of parameters, including microflora composition, intestinal permeability, epithelial structure, fecal enzymes, bacterial activity, and intestinal immunity^34^.

Rice production does not end in the field. Newly harvested rice is not sold immediately but stored for months before it is consumed. However, biosafety of *Bt*-transgenic rice to animals in the storehouse has not been well studied. Various stored pests can cause severe damage to the storage rice^35,36^. Among them, the maize weevil, *Sitophilus zeamais* is a common pest of grain storage world widely^37^. It attacks stored cereal products, including maize, wheat, rice, sorghum, oats, barley, as well as other types of stored, processed cereal products such as pasta, cassava^38^. Because of its high reproductive capacity and strong infectivity, it is considered to be the most harmful pest in grain storage^39,40^. *Theocolax elegans* is a parasitoid wasp that parasitizes larvae and pupae of economically important stored-product insect pests^41^. In this paper, we studied the effects of transgenic *Bt* stored rice (*Bt cry1Ab/cry1Ac*) on the non-target coleopteran storage pest *S*. *zeamais* and its natural enemy *T*. *elegans*.

## Results

### Effects of *Bt* rice on the maize weevil *S*. *zeamais*

We first assessed the effects of *Bt* rice on the survival and development of *S*. *zeamais* immature stages. As shown in Table 1, when *S*. *zeamais* were reared on non-GM rice under the conditions of 28 ± 1 °C, 80% relative humidity, and total darkness, the final survival rate during the developmental stages from egg to nonage was about 82% and the developmental time for this period was about 73 days. These numbers were stable from the 1^st^ to 25^th^ generation and no significant difference was detected when comparing the beetles feeding on non-GM rice or *Bt* rice (Table 1, *p* > 0.05), indicating that *Bt* rice does not affect *S*. *zeamais* development.

**Table 1:** Effect of *Bt* rice on survival and development of *S*. *zeamais* immature stages.

Generation	stages	Parameter	Rice cultivars		*p-*value
			Non-GM rice	*Bt* rice	
1	Egg	Survival rate (%)	94.56 ± 2.86*	95.18 ± 2.48	0.56
		Duration (d)	8.68 ± 1.24	8.46 ± 1.58	0.41
	Larva	Survival rate (%)	92.46 ± 3.64	91.66 ± 3.85	0.95
		Duration (d)	18.84 ± 1.97	18.27 ± 2.02	0.96
	Pupa	Survival rate (%)	88.34 ± 3.91	89.26 ± 3.59	0.61
		Duration (d)	9.27 ± 1.08	9.58 ± 1.26	0.40
	Nonage	Survival rate (%)	82.25 ± 4.86	83.54 ± 5.23	0.98
		Duration (d)	35.87 ± 2.86	36.05 ± 2.13	0.68
5	Egg	Survival rate (%)	95.76 ± 3.05	95.44 ± 2.62	0.55
		Duration (d)	8.04 ± 1.82	8.52 ± 1.36	0.31
	Larva	Survival rate (%)	92.94 ± 2.89	92.13 ± 3.57	0.51
		Duration (d)	19.26 ± 1.96	18.53 ± 2.19	0.24
	Pupa	Survival rate (%)	87.92 ± 3.64	88.06 ± 2.93	0.47
		Duration (d)	9.46 ± 1.17	8.94 ± 1.44	0.49
	Nonage	Survival rate (%)	82.42 ± 4.67	82.76 ± 4.56	0.17
		Duration (d)	36.23 ± 2.55	35.75 ± 3.02	0.58
10	Egg	Survival rate (%)	96.38 ± 2.14	96.12 ± 2.62	0.66
		Duration (d)	7.98 ± 1.86	8.26 ± 1.64	0.76
	Larva	Survival rate (%)	90.95 ± 4.21	91.86 ± 3.86	0.50
		Duration (d)	18.71 ± 2.08	19.02 ± 2.43	0.80
	Pupa	Survival rate (%)	88.81 ± 3.76	87.72 ± 3.53	0.96
		Duration (d)	9.12 ± 1.33	9.28 ± 1.51	0.67
	Nonage	Survival rate (%)	83.18 ± 4.73	82.48 ± 4.28	0.81
		Duration (d)	36.19 ± 2.08	36.47 ± 2.64	0.64
15	Egg	Survival rate (%)	95.64 ± 2.76	96.44 ± 1.96	0.27
		Duration (d)	8.06 ± 1.66	8.42 ± 1.72	0.91
	Larva	Survival rate (%)	93.47 ± 3.03	92.49 ± 2.67	0.88
		Duration (d)	18.79 ± 2.75	19.04 ± 2.37	0.90
	Pupa	Survival rate (%)	90.22 ± 3.87	88.49 ± 3.12	0.59
		Duration (d)	8.86 ± 1.59	9.07 ± 1.37	0.69
	Nonage	Survival rate (%)	82.87 ± 4.62	82.16 ± 4.32	0.91
		Duration (d)	35.86 ± 2.96	35.28 ± 2.37	0.86
20	Egg	Survival rate (%)	94.98 ± 2.94	95.40 ± 2.24	0.81
		Duration (d)	8.56 ± 1.48	8.36 ± 1.54	0.91
	Larva	Survival rate (%)	93.09 ± 3.51	92.43 ± 2.69	0.67
		Duration (d)	18.41 ± 2.63	18.59 ± 2.14	0.74
	Pupa	Survival rate (%)	87.91 ± 4.03	89.59 ± 3.92	0.89
		Duration (d)	9.34 ± 1.45	9.23 ± 1.06	0.43
	Nonage	Survival rate (%)	83.02 ± 4.81	82.86 ± 4.53	0.92
		Duration (d)	36.12 ± 2.81	35.74 ± 2.29	0.78
25	Egg	Survival rate (%)	95.46 ± 2.68	95.56 ± 2.36	0.48
		Duration (d)	8.44 ± 1.32	8.18 ± 1.52	0.54
	Larva	Survival rate (%)	92.58 ± 2.87	91.86 ± 2.98	0.86
		Duration (d)	19.07 ± 2.73	18.83 ± 2.16	0.76
	Pupa	Survival rate (%)	88.64 ± 3.65	88.51 ± 3.26	0.71
		Duration (d)	9.48 ± 1.53	9.19 ± 1.48	0.92
	Nonage	Survival rate (%)	82.81 ± 4.29	82.53 ± 4.74	0.92
		Duration (d)	35.82 ± 2.77	35.49 ± 2.23	0.59

Note: *All values are means ± standard deviations.

We then examined the effects of *Bt* rice on the longevity and fecundity of *S*. *zeamais* adults. When reared on non-GM rice under the experimental conditions, the *S*. *zeamais* adults lived 107.18 ± 8.74 days, oviposition period was 85.64 ± 5.26 days, and each female laid 276.34 ± 12.92 eggs. The life span, oviposition period, and total number of eggs laid by each female were all comparable among beetles of different generations and between beetles feeding on non-GM rice and *Bt* rice (Table 2, *p* > 0.05), suggesting that no obvious effect of *Bt* rice on the longevity and fecundity of *S*. *zeamais* adults was observed.

**Table 2:** Effect of *Bt* rice on longevity, oviposition period and fecundity of *S*. *zeamais* adults.

Generation	Parameter	Rice cultivars		*p-*value
		Non-GM rice	*Bt* rice	
1	Longevity (d)	107.18 ± 8.74*	111.26 ± 9.67	0.96
	Oviposition period (d)	85.64 ± 5.26	86.72 ± 4.98	0.87
	Fecundity**	276.34 ± 12.92	281.17 ± 12.36	0.79
5	Longevity (d)	109.53 ± 9.51	105.45 ± 8.92	0.83
	Oviposition period (d)	86.22 ± 6.82	87.19 ± 5.25	0.60
	Fecundity	275.49 ± 11.33	273.51 ± 10.94	0.88
10	Longevity (d)	108.76 ± 8.36	107.64 ± 9.27	0.34
	Oviposition period (d)	84.96 ± 6.34	85.17 ± 5.67	0.37
	Fecundity	279.26 ± 12.45	275.38 ± 11.41	0.88
15	Longevity (d)	112.71 ± 9.94	109.34 ± 7.69	0.50
	Oviposition period (d)	83.93 ± 6.84	86.03 ± 5.43	0.52
	Fecundity	272.58 ± 13.58	278.43 ± 10.27	0.41
20	Longevity (d)	106.83 ± 8.19	108.39 ± 9.64	0.81
	Oviposition period (d)	85.11 ± 5.69	86.25 ± 5.31	0.88
	Fecundity	280.11 ± 12.37	274.19 ± 10.59	0.85
25	Longevity (d)	108.47 ± 8.35	110.51 ± 7.97	0.85
	Oviposition period (d)	87.04 ± 5.48	85.49 ± 6.07	0.80
	Fecundity	272.97 ± 11.67	279.65 ± 12.62	0.71

Note: *All values are means ± standard deviations.

**Fecundity is presented by number of eggs laid by each female in 10 days starting from the first egg-laying.

### Effects of *Bt* rice on the parasitoid wasp *T*. *elegans*

*Bt* rice does not affect the development, longevity and fecundity of *S*. *zeamais*. Whether does it affect the parasitoid wasp of the maize weevil, *T*. *elegans*? We compared the developmental time, body size, and sex ratio of *T*. *elegans* feeding on non-GM rice or *Bt* rice. When *T*. *elegans* parasitized on the *S*. *zeamais* feeding on non-GM rice, the wasps took about 44 days to develop from eggs to nonage; the body length of female and male wasps was 1.46 ± 0.13 mm and 1.29 ± 0.12 mm, respectively; and female/male ratio was 4.02 ± 0.37. In all of these biological characteristics, no obvious difference was detected between *T*. *elegans* that parasitized the *S*. *zeamais* feeding on non-GM rice and *T*. *elegans* that parasitized the *S*. *zeamais* feeding on *Bt* rice (Table 3, *p* > *0*.*05*).

**Table 3:** Effects of *S*. *zeamais* feeding on *Bt* rice on development, adult body size and sex ratio of *T*. *elegans*.

Parameter		Hosts of *T*. *elegans*		*p-*value
		*S*. *zeamais* feeding on non-GM rice for 25 generations	*S*. *zeamais* feeding on *Bt* rice for 25 generations	
Duration (d)	Egg	5.62 ± 0.87*	5.54 ± 0.72	0.81
	Larva	7.47 ± 1.07	7.81 ± 0.93	0.86
	Pupa	9.25 ± 1.02	9.13 ± 1.14	0.82
	Nonage	21.56 ± 1.14	21.97 ± 1.27	0.53
Body length (mm)	♀	1.46 ± 0.13	1.42 ± 0.17	0.55
	♂	1.29 ± 0.12	1.28 ± 0.15	0.67
Sex ratio (♀/♂)		4.02 ± 0.37	4.11 ± 0.29	0.70

Note: *All values are means ± standard deviations.

### Determination of *Bt* Cry protein in the stored rice, the maize weevil and the parasitoid wasp

As we did not detect any significant influence of *Bt* rice on *S*. *zeamais* and *T*. *elegans*. We were wondering if *Bt* Cry protein exists in the *Bt* rice, *S*. *zeamais* and *T*. *elegans*. To address this question, we examined *Bt* Cry protein in all three players in the tritrophic relationship, the stored *Bt* rice, the stored-product pest *S*. *zeamais*, and the parasitoid wasp *T*. *elegans*. As shown in Table 4, we found that *Bt* Cry1Ab/Cry1Ac protein did exist in the *Bt* rice, the maize weevil larvae, and the digestive tract of maize weevil adults. But no *Bt* Cry1Ab/Cry1Ac protein was detected in the maize weevil adult tissues other than digestive tract. 10 days after the maize weevils were transferred from *Bt* rice to non-GM rice, *Bt* Cry1Ab/Cry1Ac protein disappeared from the maize weevil adult digestive tract. Moreover, *Bt* Cry1Ab/Cry1Ac protein was not detectable in the parasitoid wasp *T*. *elegans*.

**Table 4:** Determination of (*Bt Cry1Ab/Cry1Ac*) protein.

Material	*Bt Cry1Ab/Cry1Ac* protein
*Bt* rice	+
Non-GM rice	−
*S*. *zeamais*–Eggs (25^th^ generation)	−
*S*. *zeamais*–Larvae (25^th^ generation)	+
*S*. *zeamais*–Pupa (25^th^ generation)	−
*S*. *zeamais*–Adult digestive tract (25^th^ generation)	+
*S*. *zeamais*–Adult without digestive tract (25^th^ generation)	−
*S*. *zeamais*–Adult digestive tract (10 days after transferring to non-GMO rice)	−
*S*. *zeamais*–Adult without digestive tract (10 days after transferring to non-GMO rice)	−
*T*. *elegans*–Adult	−

Note: +, *Bt* protein exists; −, Bt protein does not detectable.

## Discussion

Risk assessment should be carried out when the genetically modified plants (GM plants) are cultured, stored, transported, disposed of or used^42,43^. However, most non-target risk assessments of GM rice are mainly focused on the field ecosystems, whereas coarse rice, the final product of rice production, is usually stored for a long period of time before consumption. Therefore, we focused our study on the risk assessment of *Bt* rice to the non-target organisms during storage.

In any ecosystem, there is usually a high number of non-target organism species exposing to the GM plants. There are several criteria suggested for species selection to conduct risk assessment for GM plants, for example, selecting species that are representative of their genera and/or of particular functional groups, including herbivores, pollinators, natural enemies (predators and parasitoids) of pest organisms, and decomposers of plant material^9,43^. Based on these criteria, extensive investigations were performed in the field ecosystems to assess the potential risk of *Bt*-rice to non-target herbivore pests-planthoppers^13–15^, their predators-spiders^15,20^, and their parasitoids-wasps^18,44,45^. As the maize weevil is one of the most important stored pests of rice, we evaluated the effects of *Bt* rice on the tritrophic interaction of the stored rice-*S*. *zeamais*-*T*. *elegans*. We found that *Bt* rice expressing *Bt* Cry1Ab/Cry1Ac fusion protein has no adverse effect on the growth and development of the maize weevil *S*. *zeamais*, as well as that of the parasitoid wasp *T*. *elegans*. When comparing the beetles reared on *Bt* rice and non-GM rice, there is no significant difference in all biological traits observed, including developmental speed, survival rate, and adult longevity and fecundity (Tables 1 and 2). Similarly, when *T*. *elegans* parasitized *S*. *zeamais* feeding on *Bt* rice and non-GM rice, no significant difference was found in the development, adult body sizes and female/male ratio (Table 3). These results are not due to lack of *Bt* Cry1Ab/Cry1Ac protein in the stored *Bt* rice and the maize weevils. On the contrary, we detected *Bt* Cry1Ab/Cry1Ac protein existing in the stored *Bt* rice and in the *S*. *zeamais* larvae or adults that feed on *Bt* rice (Table 4).

These findings are consistent with the results of several investigations in the rice field. For example, when feeding on Cry1 or Cry2-expressing rice pollen, non-target coleopteran species *Micraspis discolor* and *Propylea japonica* are not affected. The adult survival, longevity, and fecundity of these beetles are not different when they feed on *Bt* rice or non-*Bt* rice pollen^46,47^. Similarly, *Bt* rice is not harmful to the parasitoid wasps of non-target insects in the rice field. *Anagrus nilaparvatae* and Pseudogonatopus flavifemur are two parasitoid wasp species of rice planthoppers. Previous researches indicate that no significant difference on the development, survival, longevity, fecundity, prey consumption, and population dynamics of these parasitoid wasps is detected between the *Bt* and non-*Bt* rice treatments^18,44^. Furthermore, when *A*. *nilaparvatae* is exposed to *Bt* proteins by feeding on planthopper *N*. *lugens* honeydew produced from *Bt* rice, the development, longevity, emergence rate and fecundity of the wasp is not affected, too^45^.

When considering the biosafety of *Bt* crops, one big concern is the long-term effects of *Bt* toxin. Most of the risk assessment of *Bt* rice to the non-target organisms have been conducted within a relative short period of time and limited generations or production cycles. In this study, we evaluated the biosafety of *Bt* rice grain to the non-target organisms in the storehouse, including a storage pest and its natural enemy. Our results show that, under the laboratory trials of five years and 25 generations, *Bt* rice expressing Bt Cry1Ab/Cry1Ac fusion protein does not pose a risk to the maize weevil *S*. *zeamais* and its parasitoid *T*. *elegans*.

## Materials and Methods

### Plant material

Two cultivars of brown hybrid rice were used in this study: non-genetically modified Xinliangyou 6 rice (hereinafter referred to as non-GM rice) and *Bt*-transgenic Xinliangyou 6 rice that harbours a fused *Bt* transgene (*cry1Ab/cry1Ac*) (hereinafter referred to as *Bt* rice) (Provided by the College of Life Sciences, Huazhong Agricultural University, Hubei Province, P. R. China).

### Insect species

The maize weevil *S*. *zeamais* beetles were from a laboratory colony rearing on wheat at 28 ± 1 °C, 80% relative humidity, and under total darkness. *T*. *elegans* colony was collected from the parasitized *S*. *zeamais* in a cereal storehouse and maintained at 28 ± 1 °C, 60% relative humidity, and 12 L:12D photoperiod.

### *S*. *zeamais* development

*S*. *zeamais* adults reared on wheat were transferred to a jar containing non-GM brown rice or *Bt* brown rice for 24 hours. Then, the beetles were removed and the brown rice grains with the egg granules were put into a new jar, which was counted as the 1^st^ generation of beetles on rice. The 1^st^ generation adult beetles reared on non-GM brown rice or *Bt* brown rice were transferred into new jars containing non-GM brown rice or *Bt* brown rice, respectively, and removed 24 hours later to propagate the 2^nd^ generation of beetles on rice. This procedure was repeated thereafter until 25^th^ generation.

For the 1^st^, 5^th^, 10^th^, 15^th^, 20^th^, and 25^th^ generation of *S*. *zeamais* reared on non-GM brown rice or *Bt* brown rice, 100 brown rice grains that each carried one maize weevil egg were picked up and reared in a separate jar. During the experiment, new brown rice grains of non-GM or *Bt* rice were added into the jars when it is necessary. Development of the maize weevils were closely monitored for the stadium and survival rate of each developmental stages, such as egg, larva, pupa, and nonage. Nonage is referred to the developmental stage from eclosion to the first egg-laying of the female adults. All experiments were conducted in six replicates, so that each of them started from 600 maize weevil eggs. Means ± standard deviations are presented in the tables. T-tests were used to analyze the data on *Bt* rice and the conventional control (SAS, version 8.1).

### *S*. *zeamais* adult longevity, oviposition period and fecundity

For the 1^st^, 5^th^, 10^th^, 15^th^, 20^th^, and 25^th^ generation of *S*. *zeamais* reared on non-GM brown rice or *Bt* brown rice, paired male and female adults were put into separate bottles immediately after the eclosion. Their longevity and oviposition period were recorded. Number of eggs laid by individual female in the first 10 days of its oviposition period was used as an indicator for its fecundity. 100 pairs of male and female adults with six replicates were examined in each experiment. Means ± standard deviations are presented in the tables. T-tests were used to analyze the data on *Bt* rice and the conventional control (SAS, version 8.1).

### *T*. *elegans* development, adult body size and sex ratio

100 non-GM brown rice or *Bt* brown rice grains that bore 4^th^ instar larvae of 25^th^ generation *S*. *zeamais* were placed in a petri dish, which was put in a round sealed box (20 cm in diameter and 10 cm in height) with vent holes at the top. 24 pairs of *T*. *elegans* wasps collected from the wheat-*S*. *zeamais*-*T*. *elegans* tritrophic system were released into the sealed box, allowing the parasitoid wasps to select their hosts freely. Six repeats were set up for both non-GM brown rice and *Bt* brown rice. Parasitoid wasps were expelled 24 hours later and this time was counted as the starting point of the *T*. *elegans* development. Development of *T*. *elegans* was closely monitored by randomly dissecting *S*. *zeamais* larvae or pupae until the emergence of all the *T*. *elegans*. Body size of *T*. *elegans* adults was measured under microscope. Male and female ratio was calculated. Means ± standard deviations are presented in the tables. T-tests were used to analyze the data on *Bt* rice and the conventional control (SAS, version 8.1).

### *Bt* Cry1Ab/Cry1Ac protein detection

*Bt* Cry protein was examined using a rapid *Bt* Cry1Ab/Cry1Ac protein test kit produced by Chongqing Jinbiao Biotech Company Ltd (Chongqing, P. R. China). 20 gm of *Bt* rice grains or pooled *S*. *zeamais* and *T*. *elegans* tissues were homogenized in the Eppendorf tubes with 200 µL lysis buffer provided by kit. Paper strips for *Bt* Cry protein test were put into the sample tubes to detect *Bt* Cry1Ab/Cry1Ac protein by immune-affinity chromatography assay according to the instruction manual of the kit. Each assay was repeated for three times.

### Compliance with Ethical Standards

Ethical approval: This article does not contain any studies with human participants performed by any of the authors.

## References

[1] Yadavalli V, Neelam S, Rao ASVC, Reddy AR, Subramanyam R (2012). Differential degradation of photosystem I subunits under iron deficiency in rice. J Plant Physiol.

[2] Muralidharan K, Pasalu IC (2006). Assessments of crop losses in rice ecosystems due to stem borer damage (Lepidoptera: Pyralidae). Crop Prot.

[3] Saha P (2006). Transgenic rice expressing Allium sativum leaf lectin with enhanced resistance against sap-sucking insect pests. Planta.

[4] Sæther OA, Wang X (2000). First record of the genus paratrissocladius zavřel from the oriental region (Diptera: Chironomidae). Tijdschr voor. Entomol.

[5] Chestukhina GG (1990). Subdomain organization of Bacillus thuringiensis entomocidal proteins’ N-terminal domains. J Protein Chem.

[6] Malone, L. A., Gatehouse, A. M. R. & Barratt, B. I. P. 2008. Beyond Bt: Alternative Strategies for Insect-Resistant Genetically Modified Crops, In: Romeis, J., Shelton, A. M., Kennedy, G. G. (Eds), *Integr Insect-Resistant Genet Modif Crop within IPM Programs*. Springer Netherlands. Germany. **5**, 357–417, 10.1007/978-1-4020-8373-0_13 (2008).

[7] Meng J (2016). No impact of transgenic cry1C rice on the rove beetle Paederus fuscipes, a generalist predator of brown planthopper Nilaparvata lugens. Sci Rep.

[8] Huang J, Hu R, Rozelle S, Pray C (2005). Plant science: Insect-resistant GM rice in farmers’ fields: Assessing productivity and health effects in China. Science (80-).

[9] Romeis J (2008). Assessment of risk of insect-resistant transgenic crops to nontarget arthropods. Nature Biotechnology.

[10] Devos Y (2015). Optimising environmental risk assessments Accounting for ecosystem services helps to translate broad policy protection goals into specific operational ones for environmental risk assessments. J Cit Reports.

[11] Li Y (2017). Bt rice in China — focusing the nontarget risk assessment. Plant Biotechnol J.

[12] Yang H (2017). Review: biosafety assessment of Bt rice and other Bt crops using spiders as example for non-target arthropods in China. Plant Cell Reports.

[13] Chen M (2006). Field Assessment of the Effects of Transgenic Rice Expressing a Fused Gene of cry1Ab and cry1Ac from Bacillus thuringiensis Berliner on Nontarget Planthopper and Leafhopper Populations. Environ Entomol.

[14] Mannakkara A, Niu L, Ma W, Lei C (2013). Zero effect of Bt rice on expression of genes coding for digestion, detoxification and immune responses and developmental performances of Brown Planthopper Nilaparvata lugens (Stål). J Insect Physiol.

[15] Niu L (2017). Transgenic Bt rice lines producing Cry1Ac, Cry2Aa or Cry1Ca have no detrimental effects on Brown Planthopper and Pond Wolf Spider. Sci Rep.

[16] Bai YY, Yan RH, Ye GY, Huang FN, Cheng JA (2010). Effects of Transgenic Rice Expressing Bacillus thuringiensis Cry1Ab Protein on Ground-Dwelling Collembolan Community in Postharvest Seasons. Environ Entomol.

[17] Li Y, Romeis J, Wang P, Peng Y, Shelton AM (2011). A comprehensive assessment of the effects of BT cotton on Coleomegilla maculata demonstrates no detrimental effects by CRY1AC and CRY2AB. PLoS ONE.

[18] Tian J-C (2017). Assessing the effects of Cry1C rice and Cry2A rice to Pseudogonatopus flavifemur, a parasitoid of rice planthoppers. Sci Rep.

[19] Wang Q (2017). Combined influence of Bt rice and rice dwarf virus on biological parameters of a non-target herbivore, Nephotettix cincticeps (Uhler) (Hemiptera: Cicadellidae). PLoS One.

[20] Wang J (2017). Transcriptomic response of wolf spider, Pardosa pseudoannulata, to transgenic rice expressing Bacillus thuringiensis Cry1Ab protein. BMC Biotechnol.

[21] Li Y, Wu K, Zhang Y, Yuan G (2007). Degradation of Cry1Ac protein within transgenic Bacillus thuringiensis rice tissues under field and laboratory conditions. Environ Entomol.

[22] Crecchio C, Stotzky G (1998). Insecticidal activity and biodegradation of the toxin from Bacillus thuringiensis subsp. Kurstaki bound to humic acids from soil. Soil Biol Biochem.

[23] Saxena D, Stotzky G (2001). Bacillus thuringiensis (Bt) toxin released from root exudates and biomass of Bt corn has no apparent effect on earthworms, nematodes, protozoa, bacteria, and fungi in soil. Soil Biol Biochem.

[24] Liu Y, Li J, Luo Z, Wang H, Liu F (2016). The fate of fusion Cry1Ab/1Ac proteins from Bt-transgenic rice in soil and water. Ecotoxicol Environ Saf.

[25] Li G, Wang Y, Liu B, Zhang G (2014). Transgenic Bacillus thuringiensis (Bt) rice is safer to aquatic ecosystems than its non-transgenic counterpart. PLoS One.

[26] Liu Q (2017). Effects of 60-Week Feeding Diet Containing Bt Rice Expressing the Cry1Ab Protein on the Offspring of Inbred Wuzhishan Pigs Fed the Same Diet. J Agric Food Chem.

[27] Wang JM (2014). Influence of transgenic rice expressing a fused Cry1Ab/1Ac protein on frogs in paddy fields. Ecotoxicology.

[28] Zhu HJ (2015). A 90 Day Safety Assessment of Genetically Modified Rice Expressing Cry1Ab/1Ac Protein using an Aquatic Animal Model. J Agric Food Chem.

[29] Schrøder M (2007). A 90-day safety study of genetically modified rice expressing Cry1Ab protein (Bacillus thuringiensis toxin) in Wistar rats. Food Chem Toxicol.

[30] Wang Y (2013). Evaluation of the potential effect of transgenic rice expressing Cry1Ab on the hematology and enzyme activity in organs of female Swiss rats. PLoS One.

[31] Song H (2015). A 90-day subchronic feeding study of genetically modified rice expressing Cry1Ab protein in Sprague–Dawley rats. Transgenic Res.

[32] Zhao K (2016). Edible safety assessment of genetically modified rice T1C-1 for Sprague Dawley rats through horizontal gene transfer, allergenicity and intestinal microbiota. PLoS One.

[33] Mao J (2016). A 52-week safety study in cynomolgus macaques for genetically modified rice expressing Cry1Ab/1Ac protein. Food Chem Toxicol.

[34] Yuan Y (2013). Effects of genetically modified T2A-1 rice on the GI health of rats after 90-day supplement. Sci Rep.

[35] Arannilewa S, Ekrakene T, Akinneye J (2006). Laboratory evaluation of four medicinal plants as protectants against the maize weevil, Sitophilus zeamais (Mots). African. J Biotechnol.

[36] García M, Donadel OJ, Ardanaz CE, Tonn CE, Sosa ME (2005). Toxic and repellent effects of Baccharis salicifolia essential oil on Tribolium castaneum. Pest Management Science.

[37] Boyer S, Zhang H, Lempérière G (2012). A review of control methods and resistance mechanisms in stored-product insects. Bulletin of Entomological Research.

[38] Rugumamu CP (2012). A Technique for Assessment of Intrinsic Resistance of Maize Varieties for the Control of Sitophilus zeamais (Coleoptera: Curculionidae). TaJONAS.

[39] Hindmarsh PS, MacDonald IA (1980). Field trials to control insect pests of farm-stored maize in Zambia. J Stored Prod Res.

[40] Vásquez-Castro JA, de Baptista GC, Gadanha CD, Trevizan LRP (2012). Insecticidal Effect and Residual Action of Fenitrothion and Esfenvalerate on Sitophilus oryzae and S. zeamais (Coleoptera: Curculionidae) in Stored Maize and Wheat. ISRN Agron.

[41] Germinara GS, De Cristofaro A, Rotundo G (2009). Antennal olfactory responses to individual cereal volatiles in Theocolax elegans (Westwood) (Hymenoptera: Pteromalidae). J Stored Prod Res.

[42] EFSA Panel on Genetically Modified Organisms (GMO), Scientific Opinion on the assessment of potential impacts of genetically modified plants on non-target organisms. *EFSA Journal* **8**(11), 1877–1949. 10.2903/j.efsa.2010.1877 (2010). 10.2903/j.efsa.2010.1877 PMC13129990 42079547

[43] Prakash, D. *et al*. Risks and Precautions of Genetically Modified Organisms. International Scholarly Research Network ISRN Ecology **2011**, 369573, 13, 10.5402/2011/369573 (2011).

[44] Gao M-Q (2010). Multi-generation effects of Bt rice on Anagrus nilaparvatae, a parasitoid of the nontarget pest Nilapavarta lugens. Environ Entomol.

[45] Tian JC (2018). The rice planthopper parasitoid Anagrus nilaparvatae is not at risk when feeding on honeydew derived from Bacillus thuringiensis (Bt) rice. Pest Manag Sci.

[46] Li Y (2015). Consumption of Bt rice pollen containing Cry1C or Cry2A does not pose a risk to Propylea japonica (Thunberg) (Coleoptera: Coccinellidae). Sci Rep.

[47] Zhou X (2016). A comprehensive assessment of the effects of transgenic Cry1Ac/Cry1Ab rice Huahui 1 on adult Micraspis discolor (Fabricius) (Coleoptera: Coccinellidae). PLoS One.

